# Elucidating Pathway and Anesthetic Mechanism of Action of Clove Oil Nanoformulations in Fish

**DOI:** 10.3390/pharmaceutics14050919

**Published:** 2022-04-22

**Authors:** Kantaporn Kheawfu, Surachai Pikulkaew, Petrine Wellendorph, Louise von Gersdorff Jørgensen, Thomas Rades, Anette Müllertz, Siriporn Okonogi

**Affiliations:** 1 Department of Pharmaceutical Sciences, Faculty of Pharmacy, Chiang Mai University, Chiang Mai 50200, Thailand; kantaporn.kheawfu@cmu.ac.th; 2 Research Center of Pharmaceutical Nanotechnology, Chiang Mai University, Chiang Mai 50200, Thailand; surachai.pikul@cmu.ac.th; 3 Department of Food Animal Clinic, Faculty of Veterinary Medicine, Chiang Mai University, Chiang Mai 50100, Thailand; 4 Department of Drug Design and Pharmacology, Faculty of Health and Medical Sciences, University of Copenhagen, 2100 Copenhagen, Denmark; pw@sund.ku.dk; 5 Department of Veterinary and Animal Sciences, Faculty of Health and Medical Sciences, University of Copenhagen, 1870 Frederiksberg, Denmark; lvgj@sund.ku.dk; 6 Department of Pharmacy, Faculty of Health and Medical Sciences, University of Copenhagen, 2100 Copenhagen, Denmark; thomas.rades@sund.ku.dk (T.R.); anette.mullertz@sund.ku.dk (A.M.)

**Keywords:** clove oil, nanoemulsion, SMEDDS, SNEDDS, GABA receptor, anesthesia

## Abstract

Clove oil (CO), an essential oil of *Syzygium aromaticum*, has been reported as an anesthetic for many fish species. However, its insoluble properties require a suitable delivery system for its application. In the present study, nanoformulations of CO as a nanoemulsion (CO-NE), a self-microemulsifying drug-delivery system (CO-SMEDDS), and a self-nanoemulsifying drug-delivery system (CO-SNEDDS) were prepared for delivering CO. Zebrafish were used as a fish model to investigate oil pathways. The result shows fluorescence spots of fluorescence-labeled CO accumulate on the gills, skin, and brain. All CO nanoformulations significantly increased penetration flux compared to CO ethanolic solution. Investigation of the anesthetic mechanism of action using a rat brain γ-aminobutyric acid subtype A (GABA_A_) receptor-binding test demonstrates that CO and its major compound, eugenol, modulate [^3^H]muscimol binding. CO-NE exhibited a concentration-dependent binding activity with an EC_50_ value of 175 µg/mL, significantly higher than CO solution in dimethyl sulfoxide. In conclusion, CO enters the fish through the skin and gills. The anesthetic mechanism of action of CO is based on modulation of [^3^H] muscimol binding to GABA_A_ receptors. Among three nanoformulations tested, CO-NE is the most effective at increasing permeability and enhancing the receptor-binding activity of the oil.

## 1. Introduction

**Figure 1:**
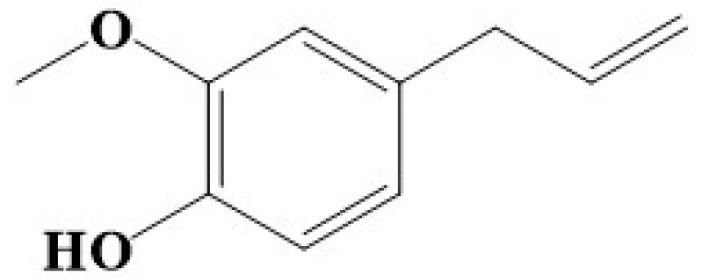
Chemical structure of eugenol.

Fish anesthetics are often used in aquaculture to ease handling and decrease physical injury to fish during farming processes such as weighing, sorting, vaccination, blood sampling, and transportation [1]. The most used fish anesthetic is tricaine methane sulphonate (MS-222) but this compound requires a long withdrawal period and has even been suspected as a carcinogen [2]. Clove oil (CO), a natural volatile oil of the clove tree (*Syzygium aromaticum* L.), has been reviewed as an alternative fish anesthetic for a variety of fish species [3,4,5]. The oil is easily extracted from fresh buds of the clove tree using simple hydro-distillation [6]. CO is normally used as a food additive and has never been reported to pose any hazard to the user and environment [7]. The absorption pathway of CO, or its main component eugenol, for fish anesthesia is unclear, while that of other anesthetics has been reported to be via the gill or skin [8]. The chemical structure of eugenol is a phenylpropanoid formally derived from guaiacol with an alkyl chain substituted *para* to the hydroxy group, as shown in Figure 1.

Although CO has the potential for anesthesia, its use in fish is limited due to its very low solubility in water. Many organic solvents, such as ethanol, isopropanol, and dimethyl sulfoxide (DMSO), are often used to increase the water miscibility of the oil. Although ethanol and isopropanol seem to be nontoxic, using large amounts of these solvents can cause severe damage to the environment due to their good solubility in water. In addition, ethanol can cause hyperactivity in fish [9,10]. DMSO is a potent organic solvent that exhibits high solubility for many polymers and hydrophobic ingredients, including essential oils [11]. However, the possibility of carcinogenesis associated with DMSO has been reported [12]. In order to circumvent the problem of water immiscibility of CO and to avoid using high concentrations of these organic solvents, we previously developed three kinds of lipid-based nanoformulations, i.e., nanoemulsions (NE), self-microemulsifying drug-delivery systems (SMEDDS), and self-nanoemulsifying drug-delivery systems (SNEDDS) as delivery systems for CO [13,14]. The extremely small droplet size of approximately 20–200 nm of NE can render the formulation highly kinetically stable even at a low surfactant concentration [15]. SMEDDS and SNEDDS are specialized forms of delivery systems containing a mixture of oil, surfactant, co-surfactant, and co-solvent. These systems can yield extremely small, dispersed oil droplets, in the micrometer or nanometer size range, after dilution with water or aqueous media [16]. The obtained results showed that the NE, SMEDDS, and SNEDDS loaded with CO (CO-NE, CO-SMEDDS, and CO-SNEDDS, respectively), could reduce anesthesia induction times to be shorter than CO ethanolic solutions (CO-EtOH) at the same CO concentration [13,14]. The anesthetic mode of action of eugenol, the major compound in CO, in fish has been reported as an activation or modulation of γ-aminobutyric acid subtype A (GABA_A_) receptors in the brain [17,18], similar to anesthetics in humans [19]. However, the pathway and mechanism of anesthetic action when CO is loaded in different kinds of nanoformulations has not yet been clarified. The present study aimed to investigate the effects of the nanoformulations on absorption and distribution pathways as well as the mechanism of action of CO in comparison with CO solutions in organic solvents for fish anesthesia. There are several mechanisms involved in fish anesthesia, e.g., blocking voltage-sensitive sodium channels [20], activating and modulating inhibitory gamma-aminobutyric acid type A (GABA_A_) receptors [21], expansion of neuronal cell membranes and, therefore, perhaps suppressing activity in the central nervous system [22]. To the best of our knowledge, the anesthetic mechanism of action of CO in fish has not been reported elsewhere. In addition, many fish anesthetics were reported to be involved in GABA_A_ receptors. Therefore, we hypothesize that the mechanism of CO may be related to these receptors. In the current study, the effect of CO and CO nanoformulations on the modulation of [^3^H]muscimol binding to GABA_A_ receptors was investigated.

## 2. Materials and Methods

### 2.1. Materials

CO was purchased from Thai-China Flavors and Fragrances Industry (Nonthaburi, Thailand). The obtained CO was analyzed using gas chromatography–mass spectrometry (GC-MS) and the method previously described [23]. Nile Red, polysorbate 20, Kolliphor EL, methanol, acetonitrile, GABA, diazepam, eugenol, and dimethyl sulfoxide (DMSO) were from Sigma-Aldrich (St. Louis, MO, USA). Captex 300 and Capmul MCM EP were kindly gifted from Abitec (Columbus, OH, USA). Ethanol and isopropanol were from VWR International (Radnor, PA, USA). Purified water was obtained from a Millipore Milli-Q Ultrapure Water purification system (Billeria, MA, USA). [^3^H]Muscimol (28.5 Ci/mmol) was from PerkinElmer (Waltham, MA, USA).

### 2.2. Animals and Housing

Fish were reared in suitable conditions [24] using a recirculating water system (Aquaschwarz, Göttingen, Germany) at 26 °C with conductivity of 550 μS. The fish were fed with live Artemia and dry pelleted feed (ZM Fish Food, Hampshire, UK) one to three times per day. Every day, ten percent of the water was refreshed. The fish were maintained at a 14 h light and 10 h darkness cycle, representing the natural environment, and were not fed for 24 h prior to testing. Twenty wild-type zebrafish (*Danio rerio*) and sixteen transparent zebrafish (tra:nac) with a significant reduction in iridophores (transparent (tra)) and an absence of melanophores (nacre (nac)), with an average length of 3.13 ± 0.21 and 2.94 ± 0.25 cm and weight of 0.37 ± 0.07 and 0.25 ± 0.09 g, respectively, were stocked in 3 L holding tanks (14 × 33 × 15 cm^3^) with a circulation water system as described above. All experiments were carried out based on permission obtained by the Animal Experiments Inspectorate (License number: 2015-15-0201-00654), the Ministry of Environment and Food, Denmark.

### 2.3. Preparation of CO Nanoformulations

CO-NE, CO-SMEDDS, and CO-SNEDDS with compositions as shown in Table 1 were formulated. For preparation of C-NE, a mixture of Tween 20 and water was firstly prepared. Then, CO was added to the mixture and stirred at 100 rpm at 40–50 °C. The obtained mixture was emulsified using a high-shear homogenizer (Ultra-Turrax T25, IKA-Werke, Staufen, Germany) at 16,000 rpm for 3 min. The pre-emulsion was then subjected to a high-pressure homogenizer (EmulsiFlex-C3, Avestin, Ottawa, ON, Canada) for 10 cycles at a pressure of 1000 bar. The obtained CO-NE was cooled down to 25 °C before use. For the preparation of CO-SMEDDS, CO was added to a mixture of Tween 20 and isopropanol. The mixture was stirred at 800 rpm at room temperature for 10 min. For the preparation of CO-SNEDDS, CO was mixed with all excipients. The obtained mixture was stirred using end-over-end rotation process for overnight at room temperature. All prepared CO nanoformulations were kept in amber glass bottles until use.

### 2.4. Particle Characterization of CO Nanoformulations

In this study, the mean droplet size and size distribution, (PDI) of all CO nanoformulations were investigated. One g of each tested formulation was diluted with 100 mL of water and gently mixed by magnetic stirring (100 rpm). Droplet size and PDI of the samples at 25 °C were determined using dynamic light scattering (DLS, Malvern Zetasizer Nano-ZS, Malvern, Worcestershire, UK) at a detecting angle of 173°.

### 2.5. CO Entering Pathways and Accumulation

In this experiment, a fluorescence oil-soluble Nile Red dye was used as a marker because CO itself does not have fluorescent properties. Detection was performed using fluorescence microscopy. A solution of 0.1% Nile Red dye in CO (CONR) was prepared by dissolving 0.1 g of Nile Red dye in 99.9 g of CO. CONR nanoformulations (CONR-NE, CONR-SMEDDS, and CONR-SNEDDS) were prepared by the same methods as the preparation for CO as described above. All CO nanoformulations with and without Nile Red dye (CONR-NE, CONR-SMEDDS, CONR-SNEDDS, CO-NE, CO-SMEDDS, and CO-SNEDDS) were added to the induction tank containing water, so that the final concentration of CO was equal to 150 mg/L. Solutions of CO-EtOH and CONR-EtOH with a CO concentration of 10% were used to compare the CO nanoformulations with and without Nile Red dye, respectively. Subsequently, the transparent zebrafish were added to these tanks for 3 min. After that, the fish were washed with distilled water and laid in a petri dish filled with normal saline solution. Fluorescence microscopy images were taken using two different light settings on a fluorescence microscope (Leica MZ FLII, Wetzlar, Germany). One setting included background light and dsRED filter light with the excitation wavelength set at 546 nm with narrow 10-nanometer passband excitation filters and an emission wavelength at 600 nm with 40-nanometer bandwidth emission filters. The other setting consisted of dsRED filter light only in order to visualize the Nile Red dye in CO formulations. The whole fish body was examined for CO transport pathways. The internal organs, i.e., intestine, spleen, liver, and brain, were separated from the fish body and investigated for possible accumulation of Nile Red dye inside the organs.

### 2.6. Skin Permeation Study

Zebrafish were randomly euthanized in an overdose of MS-222 and their skin was immediately removed. Residual fish meat was removed from the skin and the gel-like outer layer was washed out with water. The fish skin was used within 2–3 h and the skin from a single fish was used in each experiment (*n* = 3). The fish skin was placed in a modified Ussing chamber (Physiologic Instruments, Inc., San Diego, CA, USA) with slides comprising an aperture of 0.40 cm^2^. The test sample of nanoformulations was diluted in water to a CO concentration equivalent to 150 mg/L of eugenol. The exact volume of 2.0 mL of this dilution was added to the donor chamber. CO-EtOH was used as a control that had to be diluted with water to the same eugenol concentration as the test sample. The receiver chamber contained 2.0 mL of phosphate-buffered saline solution (PBS). Both chambers were maintained at 25 °C. To investigate skin permeability, each nanoformulation containing CO equivalent to 150 mg/L of eugenol was applied in the donor chamber. CO-EtOH was used as a control with the same concentration of eugenol. Aliquots of 200 µL of the receiving media were withdrawn at 1 min intervals and the media were replaced with equal volumes of PBS. The experiment was performed for 10 min. Determination of CO was performed by quantifying eugenol in CO, which was performed on an UltiMate 3000 (HPLC) system (Thermo Fisher Scientific, Waltham, MA, USA). A reverse-phase column (C18 Kinetex 5 μm (100 × 4.6 mm, Phenomenex, Torrance, CA, USA), was used as a stationary phase. HPLC conditions were set followed a previous described method [25]. Briefly, the eluent consisted of methanol: acetonitrile: water (50:25:25 *v*/*v*/*v*%), the flow rate was 1 mL/min and 20 µL of each sample was injected. The UV-detection wavelength was performed at a wavelength of 280 nm. The pure surfactant and co-solvent were run separately to determine the interference of the excipients used in the nanoformulations.

A calibration curve (peak area versus drug concentration) of eugenol was constructed by running various concentrations of eugenol in PBS. The eugenol concentration at each time point in the receiving chamber was determined using the calibration curve. Cumulative eugenol permeation (*Q_t_*) was calculated using Equation (1) [26].

(1) $$Q_{t}=V_{r}C_{t}+\sum_{i=0}^{t-1}V_{s}C_{t},$$

where *C_t_* is the eugenol concentration in the receiving chamber at each sampling time, *C_i_* is the drug concentration of the *i*th sample, and *V_r_* and vs. are the receiving solution and the sampling volume, respectively. Data are shown as cumulative drug permeation per unit of skin surface area, *Q_t_*/*S* (*S* = 0.951 cm^2^). The penetration flux (*J*) was calculated by linear regression of the experimental data using Equation (2).

(2) $$J=\frac{{\Delta Q}_{t}}{\Delta t\times S},$$

where Δ*Q_t_* is the difference in cumulative eugenol permeation between two measuring times, Δ*t* is the difference in time between two measuring points, and *S* is skin surface area.

### 2.7. Rat Brain Homogenate [^3^H]Muscimol GABA_A_ Receptor Binding

Rat cerebral cortical synaptosomes were prepared from adult male Sprague Dawley rats according to the method previously described [27] and kept at –20 °C until use. Prior to the assay, the membranes were quickly thawed in binding buffer (50 mM Tris-HCl buffer, pH 7.4), then homogenized and washed three times through pelleting with centrifugation (48,000× *g* at 4 °C). The [^3^H]muscimol binding assay was performed in 96-well microtiter plates as previously described [28]. Briefly, aliquots of membrane preparations (75–100 µg protein/aliquot) were incubated with the indicated concentrations of the test samples (e.g., eugenol at 100, 1000, 3000 µM, CO solutions in DMSO and CO nanoformulations at 10, 100, 1000 µg/mL) and [^3^H]muscimol (5 nM) in a total volume of 250 µL. Nonspecific binding was determined in the presence of 1 mM GABA, whereas diazepam (100 µM) was used as a control for positive modulation. After incubation for 1 h at 0–4 °C, the binding reaction was terminated by rapid filtration through GF/C unifilters (PerkinElmer, Waltham, MA, USA) using a 96-well Packard FilterMate cell harvester, followed by three successive washes with ice-cold binding buffer. Microscint-0 scintillation fluid (PerkinElmer, Waltham, MA, USA) was added to the dried filters, and the amount of filter-bound radioactivity was quantified in a Packard TopCount microplate scintillation counter.

Data analysis was performed using GraphPad Prism 7.0b (GraphPad Software Inc, La Jolla, CA, USA). The obtained CPM values were converted to specific binding by subtracting nonspecific binding. For the modulation curves, data were fitted with nonlinear regression analysis using Equation (3) for sigmoidal concentration response with variable slope.

Y = Bottom + (Top − Bottom)/1 + 10^(logIC^^50 − X) × Hill-Slope^, (3)

where Y is the response as % specific binding of control; X is the logarithm of the concentration; Top and Bottom refer to the upper and lower plateaus, respectively, given in the same units as Y; and log EC_50_ is the concentration giving a response halfway between Bottom and Top. Hill-Slope is the steepness of the curve. The obtained data is based on 2–3 independent experiments each with three technical replicates. All potency determinations are based on at least 3 independent experiments.

### 2.8. Statistical Analysis

Statistical tests were performed using the program SPSS 23.0 for Windows. Data from fish-skin-permeation study were tested for normality using a Kolmogorov–Smirnov test and for homogeneity of variance using a Levene test. After that, a one-way ANOVA test was used for parametric statistics. A Kruskal–Wallis ANOVA (nonparametric) test with Dunn–Bonferroni test significance level was used when requirements for parametric statistics were not met.

## 3. Results

### 3.1. Particle Characterization of CO Nanoformulations

The mean droplet sizes after 100-fold dilution of CO-NE, CO-SMEDDS, and CO-SNEDDS were 50.5 ± 0.4, 12.0 ± 1.0, and 58.2 ± 0.9 nm, respectively with PDI of 0.26 ± 0.02, 0.22 ± 0.01, and 0.07 ± 0.01, respectively. It was observed that all CO nanoformulations presented an average droplet size of less than 100 nm, indicating that addition of surfactants in CO nanoformulations could reduce droplet size and stabilize oil droplets.

In addition, the oil droplets of CO-SMEDDS with lower CO content and more surfactants than CO-NE and CO-SNEDDS were of the smallest size. It indicates that increasing surfactant concentration can reduce droplet size while adding oil concentration increases the droplet size. All nanoformulations showed low PDI values indicating monodispersed systems.

### 3.2. CO Entering Pathways and Accumulation

**Figure 2:**
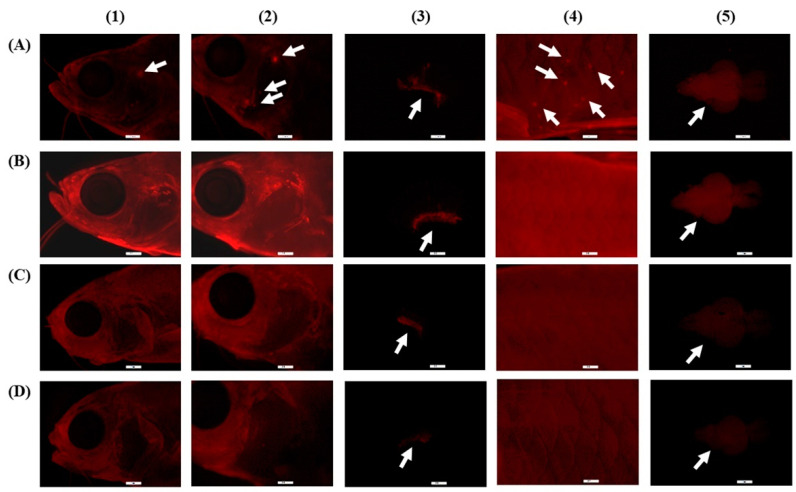
Fluorescence microscopy images of CONR-EtOH (**A**), CONR-NE (**B**), CONR-SMEDDS (**C**), and CONR-SNEDDS (**D**) accumulated in external and internal organs of transparent zebrafish: head (1), gill (2), gill filament (3), skin (4), and brain (5). These illustrations are demonstrated for qualitative determination.

Fluorescence was not observed in fish treated with CO nanoformulations without the Nile Red dye. CO labeled with Nile red dye upon contact with the fish can be clearly detected in red. As shown in Figure 2, the red spots in the fish as pointed by the white arrows indicate the location of CO with Nile Red dye. It was observed that they were located mostly in the gill and the skin of the fish. Among the test formulations, CO-NE showed the highest fluorescence intensity during external screening. To confirm this result, the relative fluorescence intensity in the fish skin and gills after exposure to the nanoformulations compared to CONR-EtOH, as a control, was calculated using Equation (4).

**Figure 3:**
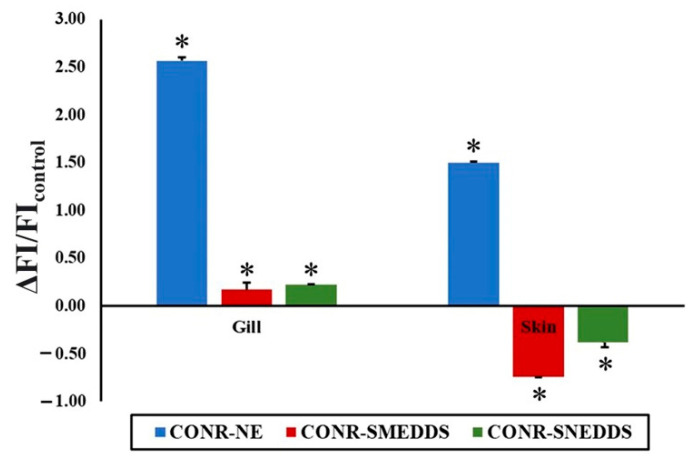
Relative fluorescent intensity of CONR nanoformulations. Asterisk (*) indicates a significant difference compared to CONR-EtOH (*p* < 0.05).

Relative fluorescence intensity = (FIsample − FIcontrol)/FIcontrol(4)

where FIsample and FIcontrol are the fluorescence intensity in fish organs after exposure to the nanoformulations and CONR-EtOH, respectively. Plotting the relative fluorescent intensity in fish skin and gills resulted in Figure 3. It was confirmed that CONR-NE showed the highest fluorescence intensity in both organs. In addition, it was found that the amount of the fluorescence in the gills was significantly higher than in the skin. This indicates the high efficiency of this nanoformulation in delivering CO. Inside the body, after examining individual organs, fluorescence accumulation was found only in the brain. These results indicate that to induce the anesthetic effects, CO enters the body of the fish through the gills and skin and then accumulates in the brain.

### 3.3. Fish Skin Permeation

**Figure 4:**
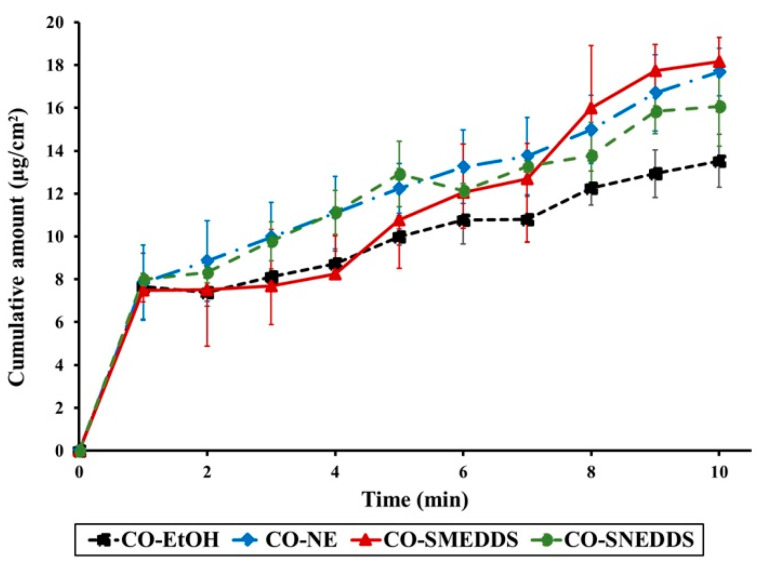
Cumulative permeation of eugenol through zebrafish skin versus time of the different CO formulations. Data are presented as means ± SD (*n* = 3).

**Figure 5:**
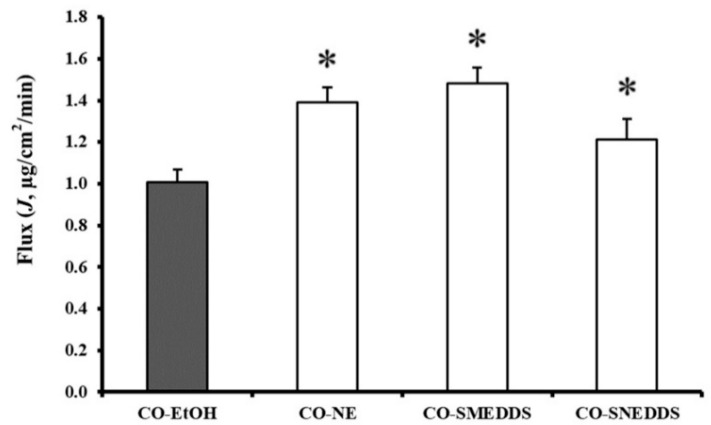
The penetration fluxes of eugenol in the different CO formulations. Significant differences (*p* < 0.05) to CO-EtOH are indicated by an asterisk (*). Data are presented as means ± SD (*n* = 3).

Zebrafish skin has previously been reviewed as a model membrane for comparing drug permeation of different preparation since its skin with collagen matrix structure appears to possess similar properties to the eye sclera [29]. In the current study, zebrafish skin is explored for the first time to determine drug permeation in the target species. Here, the permeation of CO from different formulations through zebrafish skin was compared. Eugenol was detected in the receptor media when the fish skin was treated with the various CO formulations. The cumulative eugenol permeation through fish skin for each formulation is shown in Figure 4. After 10 min, the cumulative amounts of eugenol that passed through the fish skin were found to be different depending on the formulations (13.53 ± 1.24, 17.68 ± 1.11, 18.17 ± 0.64, and 16.07 ± 1.86 µg/cm^2^ for CO-EtOH, CO-NE, CO-SMEDDS, and CO-SNEDDS, respectively). The penetration flux obtained from CO-NE, CO-SMEDDS, and CO-SNEDDS was significantly higher than that obtained from CO-EtOH as shown in Figure 5, indicating that all nanoformulations significantly enhanced the permeation of CO (*p* < 0.05).

### 3.4. GABA_A_ Receptor-Binding Assay

**Figure 6:**
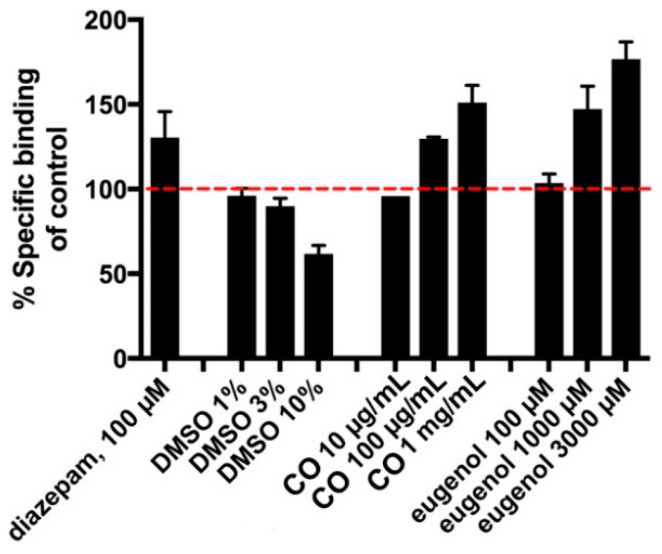
Effect of diazepam, DMSO (vehicle), CO, and eugenol on [^3^H]muscimol binding. The red line denotes normal level of total [^3^H]muscimol binding. Data are given as percentage of specific binding as means ± SD of triplicate measurements of a single representative experiment. An additional independent experiment gave similar results.

Based on a known correlation between sedative effect and positive modulation of GABA_A_ receptors [20,21,30,31,32], we further tested CO using a [^3^H]muscimol-binding assay in rat cortical homogenate. The well-known positive allosteric modulator, diazepam, was used as a positive control and was—as expected—found to increase the specific binding to approximately 130%, as shown in Figure 6. To initially test the potential GABA_A_ modulatory effect of CO, the oil was dissolved directly in DMSO to a maximal concentration of 10 mg/mL. Testing of CO-DMSO solution at the CO concentrations of 100 and 1000 µg/mL showed a concentration-dependent increase in the [^3^H]muscimol-binding level compared to normal level of total [^3^H]muscimol binding. However, the results indicated that DMSO at concentrations of 3% and above in itself produced significant inhibition of the specific binding. This precluded generation of a full curve for the pure CO and thus an accurate determination of an EC_50_ value. Eugenol in DMSO solution similarly produced clear modulation of [^3^H]muscimol binding at concentrations of 1000 and 3000 µM, indicating that the active compound in the sedative effect of CO includes eugenol. The increase in binding levels induced by both CO and eugenol reached similar or higher levels compared to diazepam. From this study, EC_50_ values of CO and pure eugenol solutions in DMSO were found to be 1 mg/mL and 1250 µM, respectively.

**Figure 7:**
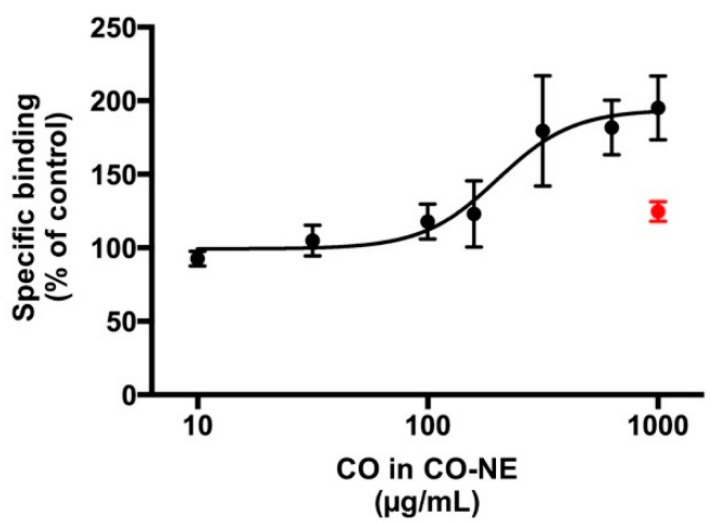
Enhancement of [^3^H]muscimol binding in the presence of CO in CO-NE. The red point denotes the CO-NE vehicle at the highest concentration tested, indicating that this did not per se significantly increase the total binding levels. Data are presented as mean percentage of specific binding ± SD for one representative curve, which was confirmed in three additional, independent experiments. Collected data for four independent repetitions gave an EC_50_ (pEC_50_ ± S.E.M.) of 175 µg/mL (3.78 ± 0.09) and an average maximal binding of 189.6 ± 4.5%.

To circumvent the problems with DMSO in determining the quantitative pharmacology of CO, the water-miscible CO nanoformulations were investigated for [^3^H]muscimol binding. It was found that vehicles of CO-SMEDDS and CO-SNEDDS inhibited binding nonspecifically (data not shown). In contrast, the CO-NE vehicle showed no interference with the levels of total [^3^H]muscimol binding. Therefore, CO-NE was chosen for further determination of EC_50_ values. CO in CO-NE showed clear modulation of [^3^H]muscimol binding in a concentration-dependent manner, as presented in Figure 7. The EC_50_ value was 175 µg/mL and the maximal effects on binding levels was 190% of the control.

## 4. Discussion

The results of this study indicate that using CO nanformulations, CO can enter into the fish though the gills and skin. It was previously reported that the pathway of this oil into the fish is only through the gills [33]. The results of the present study obviously demonstrate the potential of nanoformulations on delivering CO. The nanoformulations were able to adhere to the fish skin for a period of time that was sufficient to drive CO through the skin. Zebrafish skin has three compartments: epidermis, dermis, and hypodermis. The epidermis has mucous-secreting cells and a lateral line containing hair cells for sense movement and vibrations in the water and is covered by scales [34]. It has been reported that drug penetration was undisturbed by fish scales in the small-scale fish, plaice (*Pleuronectes platessa*) [35]. Our results demonstrate that CO nanoformulations can enhance CO penetration through fish skin compared to CO-EtOH. It is considered that the nanoformulations may encourage skin permeability by various mechanisms. Several compounds exist in CO such as terpene compounds, which may interact with fish skin by acting on the intercellular lipid structure between corneocytes to increase the fluidity of skin lipid, resulting in enhanced permeation ability [36]. Another possible mechanism is that the skin is permeated by the small-sized droplet of the nanoformulations. Our results demonstrate that CO nanoformulations presented 28 to 136 times smaller droplet sizes than CO-EtOH. This extremely small droplet size provides a large surface area that can yield a potential penetration-enhancing effect. Moreover, two surfactants, Tween 20 and Kolliphor EL, are used in the nanoformulations. Tween 20 is used in CO-NE and CO-SMEDDS, whereas Kolliphor EL is used in CO-SNEDDS. These surfactants are penetration enhancer that can effectively promote drug penetration through the skin either by altering the skin barrier or by modifying the thermodynamic activity of penetrates [37,38]. CO-SMEDDS possesses a higher concentration of surfactant than CO-SNEDDS and has an enhancing effect, allowing increased eugenol skin permeation by 1.8–5.4 fold, compared with the other CO formulations. In addition, isopropanol, used as a co-solvent of CO-SMEDDS, can perform as a skin-penetration enhancer [39]. It can alter the skin barrier and dissipate by skin absorption, thus increasing the solubility or diffusivity of drugs [40].

The highly lipophilic CO was expected to be absorbed into the lipophilic tissues of the fish body, such as fat and brain. CO indeed accumulated in the fish brain. The GABA_A_ receptor modulation found in rat brain tissue is considered to be responsible for the anesthetic effect of CO, and it is assumed that this function is conserved in the zebrafish brain. The mechanism of CO on ionotropic α1β2-GABA_A_ receptor expressed in oocytes of *Xenopus laevis* which involved with analgesic effects has been reported. Eugenol and a small amount of acetyleugenol responded the positive modulation of the GABA_A_ receptor [41]. The mechanism of eugenol action on GABA_A_ receptors has also been studied by injecting mRNAs from rat brain into *Xenopus oocytes* and measuring the increased electrophysiological response to GABA or eugenol. The results of that study suggested that eugenol can activate the GABA_A_ receptor in the central sensory system [42]. The metabolism of eugenol in fish has not been reported. However, the previous report suggests that the active metabolites of eugenol in human are eugenol itself, *cis*-and *trans*-isoeugenol, 4-hydroxy-3-methoxyphenyl-propane, 3-(4-hydroxy-3-methoxyphenyl)-propylene-1,2-oxide, 3-(4-hydroxy-3-methoxyphenyl)-propane-1,2-diol, and 3-(4-hydroxy-3-methoxy-phenyl)-propionic acid [43]. Isoeugenol is a compound structurally similar to eugenol; therefore, this compound may also potentiate GABA_A_ receptors in the same way as eugenol [32]. The result from GC-MS indicates that CO used in the present study contained 96.1% of eugenol (CO 1000 µg/mL is equal to eugenol 960 µg/mL). As mentioned above that EC_50_ values of CO and pure eugenol solutions in DMSO were 1 mg/mL and 1250 µM, respectively. Therefore, EC_50_ values of 1 mg/mL of CO solution in DMSO were calculated as nearly equal to eugenol 5800 µM. CO in DMSO solution required a 4.7- and 33.4-fold increased concentration compared to eugenol in DMSO solution and CO in CO-NE, respectively, to achieve the same effect. It may be speculated that additional compounds in CO may interact with the [^3^H]muscimol binding assay. For example, β-caryophyllene, present in CO at a level of 1.3%, was reviewed to have an anxiolytic effect via non-GABA_A_ receptors [44]. The EC_50_ value of CO in CO-NE corresponded to the concentration of commonly used CO in ethanolic solution for fish anesthesia, which is in the range of 20–150 µg/mL [33].

It is noted that vehicles or co-solvents of CO formulations, i.e., DMSO, ethanol in CO-SNEDDS, and isopropanol in CO-SMEDDS, can lead to measurable alterations in the membrane properties that might indirectly alter the function of membrane receptors and channels [45]. Additionally, DMSO, used as a solvent for CO and eugenol in the [^3^H]muscimol binding assay, causes nonspecific signals in this assay. In contrast to DMSO, CO-NE without any co-solvent but containing only a low concentration of surfactant can successfully enhance GABA_A_ binding without any nonspecific interaction. Furthermore, CO-NE can enhance the percentage of specific binding of CO at high concentrations compared to CO-DMSO. Increasing binding properties of neurotransmitter receptors with nanosized drug-delivery systems provides more effective and less toxic therapies [46]. However, CO-SMEDDS and CO-SNEDDS with a comparatively high concentration of Tween 20 and Kolliphor EL, respectively, could not be assessed under the assay conditions. In addition, the high stability of this CO-NE formulation was also previously reported [13]. Hence, a novel finding of this study is that CO-NE is the most suitable and promising nanodelivery system of CO of the [^3^H]muscimol-binding assay on GABA_A_ receptor-binding modulation.

## 5. Conclusions

Based on the data obtained from the present study, it can be concluded that the pathway into the fish of CO and CO nanoformulations is through the skin and gills, and leads to the accumulation of CO in the brain. CO-NE, CO-SMEDDS, and CO-SNEDDS can significantly enhance drug permeation compared to CO-EtOH. The [^3^H]muscimol-binding assay indicates that CO and its main compound (eugenol) are able to positively modulate GABA_A_ receptor binding. CO-NE, with a low surfactant concentration without any interference in the assay, is an appropriate delivery system of CO. The mechanism of anesthetic action of CO in CO-NE is to modulate [^3^H]muscimol binding in a concentration-dependent manner. At high concentrations, the receptor-binding effect of CO-NE was significantly higher than the CO-DMSO solution. The EC_50_ value of CO-NE corresponds to the dose of CO used for fish anesthesia. These results support our hypothesis that the main pathway for CO-induced fish anesthesia is through GABA_A_ receptors.

## Figures and Tables

**Table 1:** Composition of CO nanoformulations.

Formulations	Compositions (% *w*/*w*)							
	CO	Tween 20	Water	Ethanol	Isopropanol	Captex 300	Capmul MCM EP	Kolliphor EL
CO-NE	20	10	70	-	-	-	-	-
CO-SMEDDS	10	60	-	-	30	-	-	-
CO-SNEDDS	30	-	-	10	-	15	15	30

## Data Availability

Data are available upon request to the corresponding author.
